# Elevated plasma levels of IL-6 and MCP-1 selectively identify CML patients who better sustain molecular remission after TKI withdrawal

**DOI:** 10.1186/s13045-023-01440-6

**Published:** 2023-04-29

**Authors:** Carolina Pavlovsky, Bianca Vasconcelos Cordoba, María Belén Sanchez, Beatriz Moiraghi, Ana Varela, Rosario Custidiano, Isolda Fernandez, Maria Josefina Freitas, Maria Verónica Ventriglia, Georgina Bendek, Romina Mariano, María José Mela Osorio, Miguel Angel Pavlovsky, Ana García de Labanca, Cecilia Foncuberta, Isabel Giere, Masiel Vera, Mariana Juni, Jose Mordoh, Julio Cesar Sanchez Avalos, Gerardo Cueto, Silvia Miranda, Estrella Mariel Levy, Michele Bianchini

**Affiliations:** 1 Centro de Investigaciones Oncológicas - Fundación Cáncer (CIO-FUCA), Conesa 1003, C1426DRB Ciudad Autónoma de Buenos Aires, Argentina; 2 FUNDALEU, Buenos Aires, Argentina; 3 Hospital José María Ramos Mejía, Buenos Aires, Argentina; 4 Instituto Alexander Fleming, Buenos Aires, Argentina; 5 Hospital Posadas, Buenos Aires, Argentina; 6 Hospital Italiano, Buenos Aires, Argentina; 7 Hospital San Martín, Paraná, Argentina; 8 Hospital Italiano de Mendoza, Mendoza, Argentina; 9 Instituto de Ecología, Genética y Evolución de Buenos Aires, Buenos Aires, Argentina; 10 Instituto Alberto C. Taquini de Investigaciones en Medicina Traslacional (IATIMET, CONICET-UBA), Buenos Aires, Argentina

**Keywords:** Chronic myeloid leukemia, Treatment free remission, Cytokines, Predictive factors

## Abstract

Treatment-free remission (TFR) in chronic myeloid leukemia (CML) is safe under adequate molecular monitoring, but questions remain regarding which factors may be considered predictive for TFR. Argentina Stop Trial (AST) is a multicenter TFR trial showing that 65% of patients sustain molecular remission, and the prior time in deep molecular response (DMR) was associated with successful TFR. Luminex technology was used to characterize cytokines in plasma samples. Using machine learning algorithms, MCP-1 and IL-6 were identified as novel biomarkers and MCP-1^low^/IL-6^low^ patients showed eightfold higher risk of relapse. These findings support the feasibility of TFR for patients in DMR and MCP-1/IL-6 plasma levels are strong predictive biomarkers.

**Supplementary Information:**

The online version contains supplementary material available at 10.1186/s13045-023-01440-6.

To the editor 

Argentine Stop Trial (AST), to date, is the largest clinical trial in Latin America of chronic phase (CP)-CML patients who stopped across all types of tyrosine kinase inhibitors (TKIs) (Additional file 1: Supp Materials and Methods). Cytokine profiling has been suggested to be valuable in identifying predictive markers in other myeloid malignancies [1]; here, we measured plasma cytokine levels in 46 patients discontinuing TKIs with the aim of identifying predictive plasma biomarkers for TFR. Patients baseline characteristics and treatment information are shown in Additional file 2: Table S1. Sixteen patients (35%) lost major molecular response, leading to a molecular relapse-free survival of 65% at 36 months (Fig. 1A). Associations between TFR and prognostic variables of clinical relevance were studied, and we found significant differences only when TKI treatment duration or DMR was compared (Fig. 1B, C, D).

**Fig. 1:**
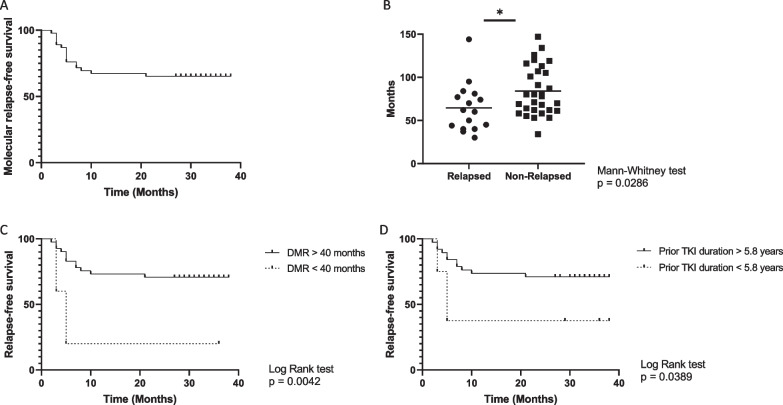
Treatment-free remission according to several variables of clinical relevance. **A** Molecular recurrence-free survival after TKI discontinuation (N = 46). The median molecular follow-up after TKI discontinuation was 31 months (range 2–38 months) overall and 34 months (range, 27 to 38 months) for the 30 patients in molecular remission without treatment. **B** Duration in months of DMR before stopping TKI in relapsed vs non-relapsed patients. **C** Molecular recurrence-free survival according to duration of stable DMR prior to TKI discontinuation (< 40 vs. > 40 months): Log-rank [Mantel-Cox] test: p = 0.004 HR = 4.29 CI: 0.64 to 28.59. **D** Molecular recurrence-free survival according to prior TKI duration (< 5.8 vs. > 5.8 years): Log-rank [Mantel-Cox] test: p = 0.039 HR = 2.80 CI 0.68–11.51)

Plasma samples from the 46 CML patients were collected at the time of stopping, when the patients were still under treatment, but all of them were already eligible for enrollment. The levels of 20 cytokines were measured (Additional file 2: Table S2) using a Luminex multiplex assay (Additional file 1: Supp Materials and Methods). To identify potential biomarkers, random forest analysis was applied and consistently identified IL-6 as the most important cytokine for TFR prediction followed by MCP-1 (Additional file 2: Table S3). Both cytokines were significantly increased in the plasma of patients in TFR compared with those who failed (Mann Whitney test P = 0.012 and P = 0.003, respectively). Furthermore, the difference in the molecular relapse-free survival between the high and low groups was also statistically significant (Additional file 2: Figure S1).

A decision tree analysis was generated by incorporating MCP-1 and IL-6 levels as the criteria to split a node, and the cut-off points were 265 pg/mL and 4.5 pg/mL, respectively (Fig. 2A). We were able to classify correctly 26 out of 30 non-relapsing patients (four false-positives) and 13 out of 15 patients who relapsed during TFR (two false-negatives). By analyzing the confusion matrix confronting these events, the model was able to predict both events with an accuracy of 87% (Fig. 2B). Moreover, we compared relapse-free survival time of the 3 terminal groups defined above; interestingly a significantly higher rate of relapse-free survival was observed in the MCP-1^low^/IL-6^hi^ and MCP-1^hi^ groups with respect the MCP-1^low^/IL-6^low^ group of patients (p < 0.0001) (Fig. 2C). By multivariate Cox proportional hazard analysis, the most relevant clinical variables, together with a combined model of MCP-1 and IL-6, were analyzed. MCP-1^low^/IL-6^low^ compared with MCP-1^hi^ showed a significant and independent predictive value for relapsed outcome (HR = 8 CI 2–36 p = 0.003) (Fig. 2D). Therefore, the MCP-1^low^/IL-6^low^ group has a negative impact on relapse-free survival, presenting eightfold higher risk of a relapse event than the MCP-1^hi^ group.

**Fig. 2:**
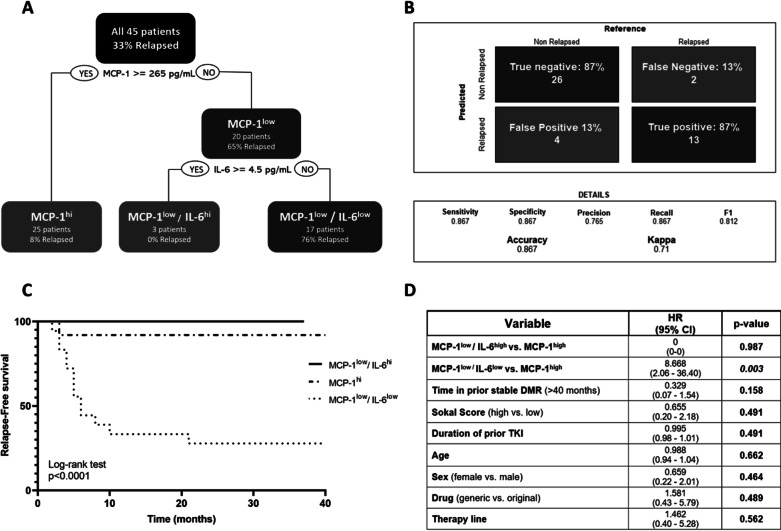
Predictive model for discontinuation. **A** Data from 30 patients on TFR who did not relapse and 15 patients who relapsed during TFR were included in a decision tree analysis by incorporating MCP-1 and IL-6 levels. This recursive partitioning showed that all patients were divided into groups according to the relapse condition. In the first partition, MCP-1 was considered the criterion; in a terminal node, MCP-1^hi^ group (> 265 pg/mL) included 25 patients, of which only 2 relapsed, while MCP-1^low^ group (< 265 pg/mL) was further split by incorporating IL-6 measurement. When MCP-1^low^ and IL-6 were combined, a relatively small percentage of patients (3/20) who did not relapse could be captured in the MCP-1^low^/IL-6^hi^ group (> 4.5 pg/mL); on the other hand, the double low group (MCP-1^low^/IL-6^low^) selectively captured those patients who lost molecular response (13 out of 17 (76%)). **B** Confusion matrix used to define the performance of a decision tree model. To estimate the power of the model, an F1-score was calculated and a value of 0.81 was determined, suggesting a very high prediction power. **C** Molecular recurrence-free survival for the MCP-1^high^, MCP-1^low^/IL-6^high^ and the MCP-1^low^/IL-6^low^ groups at the time of discontinuation. **D** Multivariate Cox proportional hazard analysis including the most relevant clinical and biological variables and IL-6/MCP-1 cytokines levels combined

Although sequential molecular monitoring is critical to rapidly detect relapse after a TKI interruption attempt [2], the accuracy prediction remains a challenge [3]. In Latin America, AST is the largest trial to date to examine TFR in patients discontinuing therapy with both branded and generic TKIs. At more than 3 years, 65% of patients maintained TFR, and similarly to Euroski trial the duration of DMR was a significant predictive factor of molecular relapse-free survival [4]. The similarity between the two trials is remarkable considering that a significant proportion of our patients were treated with generic TKIs; the higher rate of molecular remission could be attributed to the better knowledge of discontinuation criteria [5] that allowed to optimize patients enrollment and harmonized molecular monitoring available from all Argentinean molecular labs [6]. IL-6 and MCP-1 differed significantly between groups with an overall model accuracy of 87%. Our results suggest the presence of a mechanism for TKI-associated immunomodulatory effects that is distinct from a direct killing of LSC; accordingly, as suggested by Gale & Hochhause [7], it would take > 20–25 years of TKI therapy to eradicate the CML clone. Considering the function reported for both MCP-1 and IL-6 in the context of LSC regulation, they seem to play a role in favor of stem-cell proliferation [8, 9], in particular IL-6 that was identified as a mediator for STAT-3 activation. If so, how is it possible to explain our results? We speculate that in the scenario of residual CML clone, the constant pressure to proliferate by IL-6 on LSC (that does not achieve the same effect on HSC, possibly due to the selective blocking function of MCP-1) could induce LSC exhaustion [10], and consequently for patients with higher levels of these two cytokines better chances to sustain TFR. Further mechanistic and dynamics studies are required to ascertain a biological role of IL-6 and MCP-1 in CML. In the next future, by quantifying MCP-1/IL-6 plasma levels, TFR decision can be individualized according to the risk profile of the patient.

## Supplementary Information

**Additional file 1**. Supplementary Material and Method.

**Additional file 2**. Supplementary figures and tables.

## Data Availability

The datasets used and/or analyzed during the current study are available from the corresponding author on reasonable request.

## Abbreviations

TFR: Treatment free remission
TKI: Tyrosine kinase inhibitor
DMR: Deep molecular response
MCP-1: Monocyte chemoattractant protein-1
IL-6: Interleukin 6
CML: Chronic myeloid leukemia
CP: Chronic phase
AST: Argentina Stop Trial
RT-qPCR: Quantitative reverse transcription polymerase chain reaction
PB: Peripheral blood
MR: Molecular response
LSCs: Leukemic stem cells
HSC: Hematopoietic stem cell
HR: Hazard Ratio
CI: Confidence Interval
