# Supplementary material for: Elevated plasma levels of IL-6 and MCP-1 selectively identify CML patients who better sustain molecular remission after TKI withdrawal
Source: J Hematol Oncol. 2023 Apr 29;16:43. doi: 10.1186/s13045-023-01440-6 (PMC10148988; doi:10.1186/s13045-023-01440-6)
Supplement: Supplementary file 1 — Additional file 1. Supplementary Material and Method. [file 13045_2023_1440_MOESM1_ESM.docx]

**Supplementary Materials and Methods**

*Study design and participants*

For this prospective, single-arm, open-label, nonrandomized trial, we enrolled patients with CP-CML at 7 Argentinean centers in Buenos Aires city and 3 more provinces (Buenos Aires province, Mendoza and Entre Rios). Eligible patients were aged 18 years or older, had *BCR-ABL1*-positive CP-CML, and were receiving first-line or second-line (because of toxicity with first-line TKI) treatment with any of the TKI approved in our country. The minimum treatment duration with a TKI at any dose was 4 years, and the minimum duration of DMR (detectable *BCR-ABL1***^IS^** ≤ 0.01%, or undetectable *BCR-ABL1* with 10.000 or more *ABL1* transcripts, i.e. at least MR^4.0^ or better) was 2 years. To be included, patients had to show at least four RT-qPCR results showing deep molecular response (with a minimum of 3 months between studies) and no results greater than 0.01% *BCR-ABL1***^IS^** during the same period. The date of the first recording of a deep molecular response and the dates of the last three recordings of deep molecular response before TKI discontinuation were mandatory. Additionally, patients had to have records on the date of diagnosis and values for the prognostic factors in the Sokal score (i.e., age, palpable spleen size enlargement in cm below the costal margin, platelet count, and percentages of blasts, basophils, and eosinophils in peripheral blood). Pre-treatment with interferon alfa, cytarabine or hydroxycarbamide was allowed. We excluded patients with TKI failure (according to European LeukemiaNet recommendations), or atypical *BCR-ABL1* transcript (only documented e13a2 or e14a2 were admitted). In agreement with each institution’s health code, the ethics committee at each center approved the protocol. All patients were included after written informed consent was obtained.

*Molecular response assessment*

Molecular response was assessed with RT-qPCR at 2 designated standardized laboratories (Programme for harmonization to international scale) [1,2], and reported as the ratio of *BCR-ABL1* to *ABL1* on the International Scale. Molecular response assessment for *BCR-ABL1* quantification was done once a month during the first 6 months after TKI discontinuation, every 8 weeks until month 12, and then every 3 months for at least 2 years. Patients with a confirmed deep molecular response could stop TKI treatment immediately. Molecular recurrence was defined as loss of MMR, corresponding to expression of more than 0.1% *BCR-ABL1***^IS^** transcripts at any time. For patients with loss of MMR, TKI treatment was restarted with the same TKI. Particularly for the primary endpoint, molecular results were centrally reviewed by members of the study scientific committee.

*Multiplex plasma cytokine analysis by Luminex*

Blood samples were centrifuged at 400g for 30 minutes at 4°C. The upper phase was further centrifuged at 3,800g for 10 minutes and plasma samples were kept at -70°C until use. The levels of 20 cytokines, chemokines and growth factors (C, CK and GF) were measured in duplicate plasma samples by three different multiplex magnetic bead assays (Merck Millipore, MO, USA) employing a Magpix® equipment (Merck-Millipore). Measured analytes were: Eotaxin/CCL11, GM-CSF, IFNa2, IL-1a, IL-1b, IL-1Ra, IL-2, IL-4, IL-6, IL-7, IL-8/CXCL8, IL-9, IL-15, MCP-1/CCL2, TGF-α, LIF, SCF, TGF-β1, TGF-β2, TGF- β3. Each assay was performed according to the manufacturers’ instructions. Standard curves and samples were tested in duplicate. Standards were plotted and concentrations were determined using xPONENT software (version 4.2).

*Statistical analyses*

Molecular recurrence-free survival and Cox regression multivariate analysis were performed in Graphpad Prism (GraphPad Software. Version 9.0.2) and SPSS Statistics (Version 21, IBM Corporation), respectively. The Mann Whitney test was performed to compare variables between groups. Quantitative variables were dichotomized according to ROC curves and survival curves were compared with log rank test. P-values <0.05 were considered statistically significant.

To assess the importance of variables as possible predictors based on C, CK and GF, the random forest technique was used (Ranger package, Version 0.14.1 in R). A grid search algorithm guided by cross-validation was used in order to perform an hyperparameter optimization (Caret package, Version 6.0-93 and Tidyverse package, Version 1.3.2 in R).

To create a classification tree for the relapse outcome, the recursive partitioning method was used by applying the pre- and post-pruning technique. Thus, a base model was created by applying default parameters and values; then, pre-pruning was performed by modifying parameters to control the growth of the tree, followed by post-pruning, allowing the decision tree to grow completely. With the aim to control the tree growth, complexity parameter, cross-validation error (for each *nsplit*) and accuracy of the tree were verified. To create and prune the classification tree was used the 'Rpart' package. Version 4.1.16 and 'Caret' package. Version 6.0-93 in R. All analysis were performed in R (Version 4.1.1).

1. Ruiz MS, Sánchez MB, Vera Contreras YM, et al. Programme for Harmonization to the International Scale in Latin America for BCR-ABL1 quantification in CML patients: findings and recommendations. Clin Chem Lab Med. 2020 Nov 26;58(12):2025-2035. doi: 10.1515/cclm-2019-1283.
2. Ruiz MS, Medina M, Tapia I, et al. Standardization of molecular monitoring for chronic myeloid leukemia in Latin America using locally produced secondary cellular calibrators. Leukemia. 2016 Nov;30(11):2258-2260. doi: 10.1038/leu.2016.197.
