# Supplementary material for: Elevated plasma levels of IL-6 and MCP-1 selectively identify CML patients who better sustain molecular remission after TKI withdrawal
Source: J Hematol Oncol. 2023 Apr 29;16:43. doi: 10.1186/s13045-023-01440-6 (PMC10148988; doi:10.1186/s13045-023-01440-6)
Supplement: Supplementary file 2 — Additional file 2. Supplementary figures and tables. [file 13045_2023_1440_MOESM2_ESM.docx]

**Table S1: Patient characteristics**

|  |  | *Relapsed (n = 16)* | *Non-relapsed (n = 30)* |
| --- | --- | --- | --- |
| *Age at diagnosis: median (range)* |  | 45 (16 – 73) | 46 (31 – 71) |
| *Age at enrollment: median (range)* |  | 58 (24 - 78) | 57 (38 – 85) |
| *Gender* | **Female** | 7 | 16 |
|  | **Male** | 9 | 14 |
| *Months of TKI: median (range)* |  | 106 (52 – 244) | 135 (53 – 227) |
| *First Line TKI* | **Imatinib** | 12 | 25 |
|  | **Dasatinib** | 1 | 3 |
|  | **Nilotinib** | 3 | 2 |
| *TKI Type* | **Branded** | 11 | 24 |
|  | **Generic** | 5 | 6 |
| *Sokal risk* | **Low** | 5 | 17 |
|  | **Intermediate** | 7 | 7 |
|  | **High** | 4 | 6 |

**Table S2: Levels of cytokines in plasma samples**

|  | *Relapsed (n = 16)* | *Non-relapsed (n = 30)* | *R vs NR (P-value)* |
| --- | --- | --- | --- |
| *Eotaxin* | 148.1 (63.8 - 245.3) | 175.3 (65.6 - 379.3) | 0.404 |
| *GMCSF* | 79.5 (2.6 - 333.9) | 75.7 (0.4 - 579.273) | 0.595 |
| *IFNα2* | 125.4 (54.2 - 440.9) | 108.3 (64.7 - 153.5) | 0.886 |
| *IL-1α* | 34.2 (15.7 - 63.6) | 41.1 (17.6 - 75) | 0.189^1^ |
| *IL-1β* | 18.4 (7.9 - 32.1) | 20.4 (7.1 - 57.6) | 0.608^1^ |
| *IL-1RA* | 7.3 (3.7 - 13.8) | 7.5 (4.2 - 13.8) | 0.835^1^ |
| *IL-2* | 6.4 (1.1 - 44.3) | 2.3 (1.2 - 4.4) | 0.181 |
| *IL-4* | 3.8 (1.1 - 7.7) | 3 (1.2 - 10.8) | 0.204^1^ |
| *IL-6* | 3 (1.8 - 4.5) | 3.8 (1.8 - 5.7) | 0.012^1^ |
| *IL-7* | 10.1 (2.6 - 72.1) | 4.9 (2.5 - 9.5) | 0.224 |
| *IL-8* | 3.1 (1.1 - 7.8 | 3.2 (1.4 - 6.7) | 0.148 |
| *IL-9* | 13.3 (1.6 - 57.8) | 13.1 (4.2 - 69.1) | 0.990 |
| *IL-15* | 18.1 (10.4 - 49.4) | 16.5 (11.2 - 23.6) | 0.986 |
| *MCP-1* | 234.9 (147 - 368) | 289.9 (166 - 427.4) | 0.003^1^ |
| *TGFα* | 5.2 (2.9 - 9.7) | 28 (3.1 - 623.7) | 0.634^1^ |
| *TGF-β1* | 28432 (3830.6 - 63754.7) | 22054.4 (2679.5 - 87486.8) | 0.443 |
| *TGF-β2* | 1146.3 (460 - 3207.6) | 1021.8 (473.5 - 3441) | 0.388 |
| *SCF* | 77.7 (9.8 - 276.8) | 74.1 (9.8 - 190.8) | 0.824 |

^1^N = 45. Abbreviations: NR (non-relapsed); R (relapsed); Median concentrations in pg/mL (range) are listed. P-values were derived from Mann-Whitney test.

**Table S3: The top 10 ranked cytokines identified in the four different importance measures of the random forest analysis**

| *Ranking* | *Measure 1* | *Measure 2* | *Measure 3* | *Measure 4* |
| --- | --- | --- | --- | --- |
| 1 | IL-6 | IL-6 | IL-6 | MCP-1 |
| 2 | MCP-1 | MCP-1 | MCP-1 | IL-6 |
| 3 | IL-8 | IL-7 | TGF-β2 | IL-9 |
| 4 | IL-2 | IL-8 | IL-4 | IL-1α |
| 5 | IL-7 | Eotaxin | IL-1α | TGF-β2 |
| 6 | IL-4 | IL-1α | TGF-β1 | TGF-α |
| 7 | GMCSF | IL-4 | IL-7 | IL-2 |
| 8 | IL-9 | GMCSF | IL-9 | IL1-RA |
| 9 | TGF-β2 | IL-9 | IL-2 | IL-7 |
| 10 | IFN-α2 | IFN-α2 | IL-8 | TGF-β1 |

*Random Forest Analysis was restricted to 18 cytokines (TGF-β3 and LIF were excluded as it was consistently below the detection limit).*

*
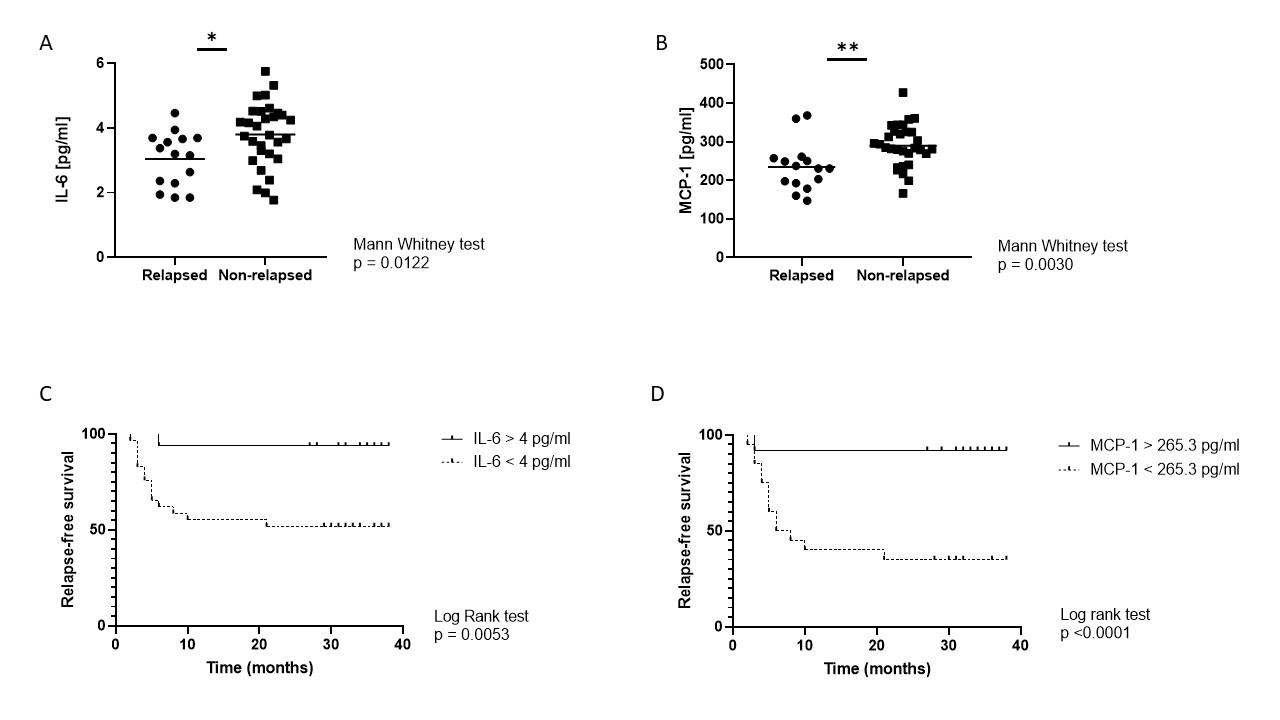
*

**Figure S1. Plasma cytokines at baseline and their prognostic relevance. (A)** Levels of circulating IL-6 (pg/mL) in plasma samples from relapsed vs non-relapsed patients at the time of discontinuation. **(B)** Levels of circulating MCP-1 (pg/mL) in plasma samples from relapsed vs non-relapsed patients at the time of discontinuation. **(C)** Molecular recurrence-free survival according to pg/mL of IL-6 in plasma samples at the time of discontinuation (at 36 months 94% vs. 52% log-rank test. p=0.0053 likelihood ratio = 1.87) **(D)** Molecular recurrence-free survival according to pg/ml of MCP-1 in plasma samples at the time of discontinuation (at 36 months MCP-1 92% vs. 35%. log-rank test p<0.0001 likelihood ratio = 3.25).
